# Dynamic surveillance of SARS-CoV-2 shedding and neutralizing antibody in children with COVID-19

**DOI:** 10.1080/22221751.2020.1772677

**Published:** 2020-06-09

**Authors:** Pengcheng Liu, Jiehao Cai, Ran Jia, Shuai Xia, Xiangshi Wang, Lingfeng Cao, Mei Zeng, Jin Xu

**Affiliations:** a Department of Clinical Laboratory, Children’s Hospital of Fudan University, Shanghai, People’s Republic of China; b Department of Infectious Diseases, Children’s Hospital of Fudan University, Shanghai, People’s Republic of China; c Key Laboratory of Medical Molecular Virology (MOE/NHC/CAMS), School of Basic Medical Sciences, Fudan University, Shanghai, People’s Republic of China

**Keywords:** Children, SARS-CoV-2, COVID-19, viral shedding, neutralizing antibody

## Abstract

Coronavirus disease 2019 (COVID-19) caused by severe acute respiratory syndrome coronavirus 2 (SARS-CoV-2) emerged in China and quickly spread globally. In this study, we investigated the characteristics of viral shedding from different sites and the neutralizing antibody (NAb) response during the acute and convalescent phases of nine children with COVID-19. SARS-CoV-2 was detected in their nasopharyngeal swabs (9/9, 100%), stool samples (8/9, 89%), and oropharyngeal swabs (3/9, 33%) but was not detected in their serum and urine samples. The median duration of viral shedding detected in nasopharyngeal swabs, oropharyngeal swabs, and stools was 13, 4, and 43 days respectively, and the maximum duration of viral shedding detected from stools was 46 days after discharge. In children, nasopharyngeal swabs appear to be a more sensitive specimen type for the diagnosis of COVID-19 compared with oropharyngeal swabs. Three of eight patients produced NAbs in the acute phase, and NAbs were detected in all eight patients with convalescent sera. The results of this study provide valuable information for the diagnosis and surveillance of COVID-19 and development of SARS-CoV-2 vaccines for use in children.

An outbreak of coronavirus disease 2019 (COVID-19) caused by severe acute respiratory syndrome coronavirus 2 (SARS-CoV-2) emerged in Wuhan, China in December 2019 [1]. As of 8 May 2020, there have been 3,759,967 confirmed cases, including 259,474 deaths, globally [2]. However, there is a lack of study on the detailed characteristics of viral shedding and neutralizing antibody (NAb) response in children with COVID-19. Here, we report a prospective follow-up investigation on viral shedding and SARS-CoV-2-specific NAb in children with COVID-19.

Nine children with COVID-19 admitted to the Children’s Hospital of Fudan University between January 19 and 14 February 2020 were enrolled. This hospital was the only designated hospital for children with COVID-19 in Shanghai, China. Clinical, epidemiological, laboratory, and radiological data from these patients were obtained from their electronic medical records.

Specimens (nasopharyngeal and oropharyngeal swabs and stool, serum, and urine samples) were collected at multiple timepoints from each patient during hospitalization and after discharge. Nasopharyngeal and oropharyngeal swabs were collected with synthetic fibre swabs by physicians and inserted into viral transport medium. Real-time reverse-transcription polymerase chain reaction (rRT-PCR) testing was performed to detect SARS-CoV-2 in the specimens in accordance with the recommended protocol. The criteria for discharge were the absence of clinical symptoms and radiological abnormalities and two consecutive negative rRT-PCR tests on respiratory specimens. For patients with persistent viral shedding in their stool after discharge, we monitored the stools continuously and collected data through 10 April 2020. Nucleic acid extracted from the positive specimens of four patients was used for whole-genome sequencing by next-generation sequencing. NAbs against SARS-CoV-2 were evaluated by pseudovirus-based neutralization assays. The 50% inhibitory concentration (IC_50_) was defined as the serum dilution at which the relative light units (RLUs) were reduced by 50% compared with the control wells without added patient serum [3].

Categorical variables are expressed as numbers (%). Continuous variables are expressed as medians (ranges), and significant differences were determined by the Mann–Whitney U test. Correlations were calculated using the Spearman correlation.

Of the nine patients, five (cases 1–5) patients had only mild upper respiratory tract infection (URTI) manifestations on admission, and the other four (cases 6–9) had chest radiographs showing evidence of pneumonia. The median age of the patients was 85 months (range, 7–139 months), and four patients (44%) were male. Cough (78%), fever (67%), and sore throat (56%) were the major symptoms. Levels of C-reactive protein, procalcitonin, and IL-6 were elevated in 4 (44%), 6 (67%), and 2 (22%) patients respectively, and lymphocyte counts were normal in all patients. No significant differences in the common laboratory results were observed between the URTI and pneumonia patients (Table S1).

The SARS-CoV-2 full genome sequences from four patients (two patients in each of the two groups) were found to be identical or nearly identical to the reference sequence (NC_045512.2). There was one variable nucleotide and one variable amino acid in case 3, seven variable nucleotides and three variable amino acids in case 5, three variable nucleotides and three variable amino acids in case 9, and no variable nucleotides in case 6.

All patients had SARS-CoV-2 shedding in their nasopharyngeal swabs (NSs). The median duration of viral shedding from the day of illness onset to the last positive nasopharyngeal swab collected as part of clinical care was 13 days (range, 6–24 days), and there was no significant difference between URTI and pneumonia patients (*p = 0.539*). Only three of nine patients had oropharyngeal swabs (OS) that were SARS-CoV-2 RNA-positive for a median duration of four days (range, 3–10 days), and all three had pneumonia (Figure 1). SARS-CoV-2 RNA was not detected in the serum or urine samples.

**Figure 1.**
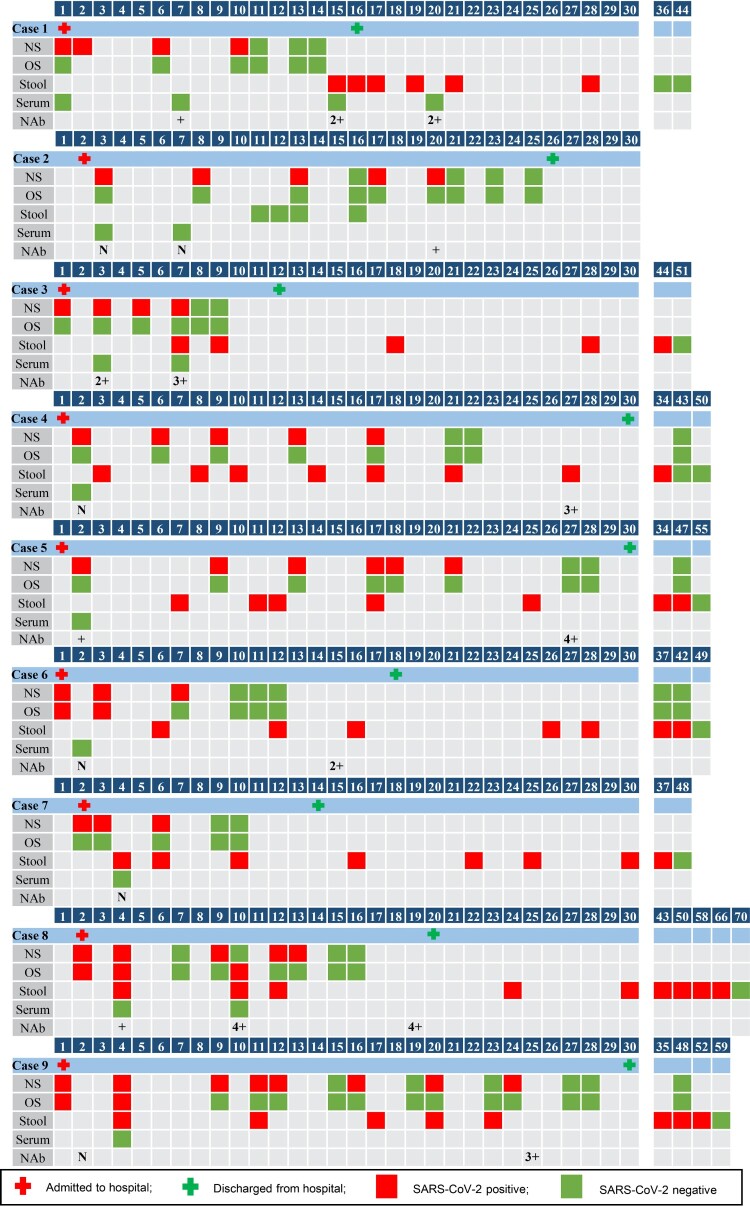
SARS-CoV-2 shedding and NAb response in children with COVID-19 according to the day of illness onset. (NS, nasopharyngeal swab; OS, oropharyngeal swab; N, IC_50_ < 80; +, IC_50_ = 80–499; 2+, IC_50_ = 500–999; 3+, IC_50_ = 1000–2000; 4+, IC_50_ > 2000).

Except for one patient who was negative for SARS-CoV-2 RNA upon hospital admission, all patients had SARS-CoV-2 shedding in their stools for a median duration of 43 days (range, 28–66 days). All eight of these patients had persistent viral shedding in their stools after discharge, and the median duration from the day of discharge to the day of last positive collected stool at follow-up was 22.5 days (range, 4–46) (Figure 1).

Among the eight patients in acute phase (1–4 days after illness onset), NAbs were produced in the sera of three patients (IC_50_ > 80) (Figure 1). However, viral shedding in the respiratory tract was still persistent in these three patients, and the viral shedding duration after NAb development was as long as 19 days in case 5. NAbs were detected in all eight patients with convalesent sera (Figure 1), and the median IC_50_ was 1,483.9 (range, 307.2–5,925.4) (Table S2). A positive correlation was observed between the duration of viral shedding in stool and the NAb titres of convalescent sera (*r *= 0.810, *p *= 0.015), whereas no correlation was observed between the duration of viral shedding in NSs and the NAb titres (*r *= 0.275, *p *= 0.509).

Oropharyngeal swabs were used much more frequently than NSs in China during the COVID-19 outbreak because they are more convenient to collect. Here, we conducted consecutive surveillance of SARS-CoV-2 RNA in nine children with COVID-19 after admission and discharge. SARS-CoV-2 was detected in all the NSs and 33% of oropharyngeal swabs, indicating that, in children, NSs appear to be a more sensitive specimen type for COVID-19 diagnosis compared with oropharyngeal swabs. A report from 353 adult patients also demonstrated a higher detection rate in NSs than in oropharyngeal swabs (19.0% *vs.* 7.6%, respectively) [4]. Thus, choosing NSs as the proper type of upper respiratory tract specimen for molecular diagnosis of COVID-19 is recommended [5,6]. One reason for the higher positive rate of SARS-CoV-2 in NSs is that the respiratory tract is the major route of SARS-CoV-2 transmission. Nasal epithelial cells, including goblet cells and ciliated cells, show the highest expression of SARS-CoV-2 entry receptor ACE2 among cells in the airway, suggesting that the nasal epithelial cells might be the first loci of original infection [7]. Furthermore, it is easier to obtain high-quality specimens from children’s nasopharynx than from their oropharynx, because oropharyngeal sampling often elicits the gag reflex and can make patients, especially children, less compliant.

The median viral shedding duration in paediatric oropharyngeal swabs was only four days, much shorter than that in adults (median, 20 days) [8], probably because paediatric patients had milder disease severity than did adult patients [9]. Here, all patients with positive oropharyngeal swabs showed evidence of pneumonia by chest radiography; however, more cases and evidence are needed to elucidate the relationship between oropharynx virus excretion and disease severity. The observation that virus was still detected in NSs after oropharyngeal swab results turned negative suggests that NSs might be more reliable for surveilling SARS-CoV-2 shedding in the respiratory tract.

Our findings demonstrate a much higher positive rate of virus detection in the stool samples of children (8/9, 89%) than in those of adults (27%–50%) [10,11]. A study conducted in Guangzhou showed eight of ten children with COVID-19 had SARS-CoV-2 rRT-PCR-positive rectal swabs, which is similar to our finding [12]. It is worth noting that SARS-CoV-2 shedding in the stool after discharge lasted for as long as 46 days (Case 8), which is the longest duration reported to date [13]. Live SARS-CoV-2 is present in stool samples [14], but it remains unclear whether transmission can occur via virus-contaminated faeces. The infectivity of SARS-CoV-2-positive faeces needs to be tested by virus culture and animal models. Because the current discharge criteria for COVID-19 are based on obtaining a negative viral RNA test on respiratory specimens, we should be cautious when caring for children after discharge who may have persistent viral shedding in their stools. When such children are discharged, physicians should recommend home quarantine and even virus monitoring.

We observed that three of eight patients induced NAbs in the acute phase of SARS-CoV-2 infection. However, viral shedding was persistent after NAb production, indicating that the NAbs generated in the acute phase were insufficient to clear the SARS-CoV-2 quickly and clearance of SARS-CoV-2 may require time and the development of high NAb titres.

A study conducted in 175 adult patients showed that about 30% of recovered patients generated very low NAb titres (IC_50_ < 500), with 10 patients whose NAb titres were below the limit of detection [15]. In comparison, only one case in our study generated a low NAb titre (IC_50_: 307.2) in the convalescent phase, and all other patients with convalescent sera produced medium to high NAb titres (IC_50_ > 500). Together, these findings indicate that children develop a robust NAb response after SARS-CoV-2 infection, and humoral immunity may play a more critical role in the recovery of paediatric patients than in that of adult patients.

The NAb titres in convalescent sera were also observed to have a moderate positive correlation with the duration of viral shedding in stool. The patients with longer durations of viral shedding in stool probably had higher viral loads in the intestine, which might stimulate stronger humoral immune responses against SARS-CoV-2. From another perspective, the high NAb titres during the convalescent period may not effectively shorten the duration of viral shedding in the faeces after recovery. However, given the small sample size of this study, more research is needed to clarify the correlation between NAb production and viral shedding in children with COVID-19.

In conclusion, our study demonstrates the features of SARS-CoV-2 shedding and NAb response in paediatric patients with COVID-19. The results provide valuable information for COVID-19 diagnosis and surveillance in children and for SARS-CoV-2 vaccine development.
